# Obsessional thinking and autistic traits are each uniquely associated with greater traits of gender dysphoria in clinical and nonclinical adult samples

**DOI:** 10.1186/s13229-025-00649-1

**Published:** 2025-03-17

**Authors:** Karl Mears, Dheeraj Rai, Punit Shah, Chris Ashwin

**Affiliations:** 1 Department of Psychology, Centre for Applied Autism Research, University of Bath, Bath, BA2 5LS UK; 2 Centre for Academic Mental Health, Avon and Wiltshire Partnership NHS Mental Health Trust, Bristol Medical School, University of Bristol, Bristol, UK; 3 Department of Psychology, Centre for Applied Autism Research (CAAR), University of Bath, Bath, BA2 7AY UK

**Keywords:** Autism spectrum disorder, Gender dysphoria, Obsessive compulsive disorder

## Abstract

**Background:**

Research has demonstrated a strong relationship between autism and gender dysphoria (GD) and that this relationship could be explained by obsessional interests which are characteristic of autism. However, these studies often measured obsessions using either single items which questions the reliability of the findings, or within autistic trait measures meaning the findings may simply index a more general relationship between autistic traits and GD. Therefore, the present study aimed to investigate the relationships between obsessional thoughts and traits of GD using a measure of obsessional thoughts alongside a measure of autistic traits, which was investigated in both non-clinical and clinical samples.

**Methods:**

A total of 145 non-clinical participants took part in Study 1 and all completed the Autism-Spectrum Quotient (AQ) as a measure of autistic traits, the Obsessive-Compulsive Inventory-Revised (OCI-R) obsessional thoughts subscale as a measure of obsessional thoughts, and the Gender-Identity/Gender-Dysphoria Questionnaire (GIDYQ) to measure traits of GD. For Study 2, a total of 226 participants took part in Study 2 and all completed the same measures as in Study 1. They included participants diagnosed with GD (N = 49), autism (N = 65), OCD (N = 46) and controls with no diagnosis (N = 66).

**Results:**

The hierarchical linear regression for Study 1 showed that both total AQ and OCI-R obsessional thoughts scores were uniquely associated with GIDYQ scores, with no interaction effect between the scores. The results for Study 2, from a hierarchical linear regression, once again found that obsessional thoughts and autistic traits were each uniquely associated with GIDYQ scores, but not their interaction. The GD and autistic groups both reported significantly greater traits of GD than the OCD and control groups, with the GD group reporting higher scores than the autistic group.

**Limitations:**

Participants self-reported their diagnoses for Study 2, but diagnostic tests to verify these were not administered. Traits of GD were also measured at a single point in time, despite such traits being transient and continuous.

**Conclusions:**

The results show both obsessional thoughts and autistic traits are uniquely associated with GD, and that autistic people experience greater traits of GD than other clinical groups.

**Supplementary Information:**

The online version contains supplementary material available at 10.1186/s13229-025-00649-1.

## Introduction

The Diagnostic and Statistical Manual of Mental Disorders (DSM-V) characterises autism based on difficulties in social reciprocity and communication alongside restricted interests and repetitive behaviours (RRBs) [3]. This definition has been questioned by some based on its focus on deficits [4]. In contrast, the neurodiversity model views autism as a natural variation of individual differences rather than in terms of deficits [29, 40], and focuses on autism as an identity and towards promoting positive self-identification of people as autistic and neurodiverse [34].

Identity can be defined as the way a person understands and views themself, as well as by how others view them [26]. Gender identity is how one chooses to identify based on gender norms, attitudes and behaviours within culture and society [9]. Gender diversity is a term used to describe people whose gender identities and/or expressions of gender are different from social and cultural expectations attributed to their sex assigned at birth. This may include trans, non-binary and other gender non-conforming identities or people who do not identify as cisgender, as well as many other culturally diverse identities [9]. Gender diversity does not imply experiencing distress and is not a clinical mental health condition.

The intersection between autism and gender diversity has become increasingly more established within the literature. For example, roughly 11% of gender-diverse people are autistic [31], autistic and non-autistic gender diverse people report elevated levels of autistic traits when compared to their cisgender counterpart [56], and autistic children and adolescents are more likely to endorse the wish to be the opposite sex when compared to non-autistic peers [37, 49, 55].

Some people who identify as gender diverse may also experience feelings of gender dysphoria (GD), which is a state of distress because a person’s experienced or expressed gender is different to their biological sex and produces a desire to be a different gender or biological sex [3]. GD has previously been described in the literature in two ways, with one view approaching GD as a categorical construct based on whether individuals display certain symptoms in order to meet a diagnostic criteria for GD [3]. An alternative view sees GD as a dimensional construct, with individuals demonstrating varying degrees of GD feelings that can be measured using continuous self-reported measures [12, 14, 44]. There has been increased research into the relationship between autism and gender dysphoria (GD) following international reports of an overrepresentation of autistic individuals within gender clinics [8, 13, 27, 33, 38]. Studies have reported that up to 26% of people attending gender clinics for a suspected diagnosis of GD also have a diagnosis of autism [33, 41]. These rates are notably higher than the prevalence rates of autistic children in the general population of approximately 1.76–1.85% [36, 42]. Research has also shown that people diagnosed with GD have a higher number of autistic traits compared to cisgender people [2, 47, 54]. Together, the evidence has demonstrated an association between traits of GD and higher levels of autistic traits and greater rates of autism diagnoses (see Glidden et al. [23] and Thrower et al. [50] for reviews of the literature).

While most research to date has focused on measuring the degree of autistic traits and prevalence rates of autism diagnoses within gender dysphoric samples, other research has investigated self-reported traits of GD within autistic samples. For example, George and Stokes [22] recruited autistic and non-autistic participants and measured self-reported autistic traits using the Autism-Spectrum Quotient (AQ; Baron-Cohen et al. [6]) and traits of GD using the Gender-Identity/Gender-Dysphoria Questionnaire for Adolescents and Adults (GIDYQ; Deogracias et al. [14]). Lower mean scores for the GIDYQ represent greater traits of GD. Across the entire sample, the results showed total scores for the AQ and all five of its subscales were all negatively related to mean GIDYQ scores, such that higher autistic traits were associated with greater traits of GD. The results also showed that autistic people reported greater traits of GD compared to a non-autistic sample. Those results are consistent with findings by Kallitsounaki and Williams [30], who also utilised the AQ and GIDYQ on participants from the general population, and who also found total AQ scores were negatively related with mean GIDYQ scores. Kallitsounaki and Williams [30] also found that autistic adults were approximately 50 times more likely to demonstrate clinically significant levels of GD compared to non-autistic adults. While the research has demonstrated a clear link between higher autistic traits and greater traits of GD, the mechanisms underlying this relationship are still unclear.

One explanation for the link between autistic traits and GD is the obsessive thinking style, which is one of the characteristics of autism [53, 61]. Based on this idea, some autistic people may become overly fixated on cross-sex activities and objects and develop an intense focus on topics and behaviours related to another gender, which may then manifest into characteristics of GD [53, 61]. An alternative possibility is that increased obsessive thinking in gender diverse people might lead to a presentation that mimics autistic characteristics, such as RRBs [57]. Case studies of people diagnosed with both autism and GD have highlighted the frequent occurrence of gender related thoughts, and that these may have obsessive characteristics about them [21]. A study by VanderLaan et al. [53] measured the prevalence of parent-reported obsessional interests in 3-to-12-year-old children referred to mental health services for GD, based on scores from Item 9 from the Child Behaviour Checklist (CBCL; Achenbach [1]; “Can’t get his/her mind over certain thoughts”). They reported that children who were referred for GD were rated by their parents as showing elevated rates of obsessional thoughts in comparison to non-referred children. Another study using the same CBCL item reported by teachers also found elevated rates of obsessions in young children referred for GD compared to children referred for other mental health conditions and healthy control children [61]. While the research has demonstrated a link between obsessive thoughts and traits of GD, the direction and nature of the relationship remains unclear.

While the evidence reported above has reported a relationship between obsessional thinking and greater traits of GD, there are a number of issues that need to be considered about the research. Two of the key papers reporting a link between GD and obsessions [53, 61] measured obsessional thoughts using only a single item, and it has been shown that one item measures have weaker predictive validity compared to multi-item scales [15, 43]. The authors of the two papers also concluded that the single item reflected obsessional interests which are characteristic of autism, even though the item was not designed to measure autistic characteristics. It is therefore not clear from the results using the single item if the relationship between GD and obsessional thoughts are reflective of autistic traits or obsessional thoughts in general. The use of a multi-item measure, such as the Obsessive-Compulsive Inventory-Revised (OCI-R, Foa et al. [18]), would measure obsessional thoughts associated with OCD and therefore be differentiable to the obsessional thoughts commonly reported in autistic people. The studies above have also focused on measuring obsessional thoughts and gender dysphoria in young children, as the sample in the study by Vanderlaan et al. [53] was aged from 3 to 12 and the mean age of the sample in the study by Zucker et al. [61] was 7.7 years. This means the participants were very early in social development including their gender identity, and so further evidence about the link between autistic traits and obsessional thoughts with GD is needed in older samples whose gender identities may be more developed.

The present study aimed to investigate the relationship between obsessional thoughts and autistic traits with traits of GD by utilising a multi-item measure of obsessions as part of the full OCI-R which was developed and validated for measuring OCD symptoms in diverse clinical [18, 20] and non-clinical samples [5, 48, 60], rather than autistic traits, and by recruiting adult samples. To do this we included the obsessions subscale of the revised version of the Obsessive-Compulsive Inventory (OCI-R; Foa et al. [18]), which measures obsessional thoughts characteristic of OCD differentiable to those typically seen in autism. We also included the 50-item version of the AQ to measure general autistic traits, given previous findings of an association between higher autistic traits and GD. The AQ does not include a specific subscale for measuring autistic obsessional traits and the AQ total scores and all five subscales have previously been shown to be associated with GD [22].

The main aim of the present study was to test the relationships between obsessional thoughts and autistic traits with traits of GD across two different studies with separate adult samples. Study 1 tested these relationships in a non-clinical sample, while Study 2 tested the same relationships across clinical samples. Based on previous research [22, 30, 32] it was expected that autistic traits would be associated with elevated traits of GD in both clinical and non-clinical adult samples. If obsessional thoughts are related to GD separate to the obsessions seen in autism, then it was hypothesised that obsessional thoughts would relate to GD using a measure of obsession thoughts independent of autistic traits.

## Study one

### Methods

#### Participants

A total of 187 non-clinical participants (mean age = 26.62, SD = 11.46; sex assigned at birth: 128 female/59 male) were recruited for the study. The final sample^1^ consisted of 145 participants for data analysis (mean age = 27.50, SD = 12.50; sex assigned at birth: 94 female/51 male). Participants were recruited through social media (e.g., Twitter, Facebook, Reddit etc.) and the University of X undergraduate Research Participation Scheme. Participants were eligible if they were aged 16 years or over and self-reported not having a diagnosis of any psychiatric or neurodevelopmental conditions, including autism. Within the sample, 131 participants identified as being cisgender and 14 participants identified as transgender.^2^

#### Measures

##### Autism-Spectrum Quotient (AQ; Baron-Cohen et al. [6])

The AQ is a self-report questionnaire which measures the degree of autistic traits. The questionnaire consists of 50 items which cover five domains associated with autism: social skills, communication skills, imagination, attention to detail, and cognitive inflexibility. Each item uses a four-point Likert scale with answers ranging from 0 (definitely agree) to 1 (definitely disagree) with some items being reverse scored. The AQ total scores range from 0 to 50, with values of 26 or higher indicating a high probability of meeting diagnostic criteria for autism [59].

##### Obsessive Compulsive Inventory-Revised (OCI-R; Foa et al. [18])

The OCI-R is an 18-item self-report measure involving the key DSM-IV symptoms of OCD occurring within the last month. The items use a 5-point Likert scale ranging from 0 (Not at all) to 4 (Extremely). In addition to a total score, the measure can be divided into six subscales: washing, checking, ordering, obsessing, hoarding and neutralising [28]. Each subscale consists of three items. The total score ranges from 0 to 72, and each subscale score ranges from 0 to 12. Total scores of 21 or above indicate a presence of clinical OCD [18]. The use of the OCI-R for measuring symptoms of OCD has been validated in diverse clinical [18, 20] and non-clinical samples [5, 48, 60]. The focus of the study was to measure obsessional thoughts, and not OCD symptoms more generally. Although participants completed the full OCI-R scale in the survey, we utilised the obsessions subscale in the main analyses. First, it was important to test if obsessional thoughts were related to GD and not to other symptoms of OCD, to justify our position of utilising only the obsessional thoughts subscale in the main analysis. See the Analysis section in the Results and the Supplementary Material for evidence of our justification.

##### Gender-Identity/Gender-Dysphoria Questionnaire for adolescents and adults (GIDYQ; Deogracias et al. [14])

The GIDYQ is a 27-item self-report measure of gender identity and GD. The items assess an individual’s feelings, wishes, thoughts and behaviours towards their biological sex assigned at birth and their experienced gender within a twelve-month period, with an example item “In the past 12 months have you felt unhappy about being a woman?”. The questionnaire has one version for males and one version for females, which are completed in accordance with the participants’ sex assigned at birth. Responses are recorded using a 5-point Likert scale, ranging from 1 (always) to 5 (never), such that a lower score is indicative of higher levels of GD. A mean score of 3.00 or below indicates clinically significant levels of gender dysphoria [14, 46].

#### Procedure

Participants completed the study online and Qualtrics was used to present the survey and record responses. They completed the measures in the order of the AQ, OCI-R and the GIDYQ. Participants viewed the items one item at a time and selected a single response per item on their device using a mouse click or finger press. Completion of the survey took approximately 20 minutes.

#### Analysis

##### Use of the OCI-R obsessional thoughts subscale

Part of the primary aim of this study was to measure the relationship obsessional thoughts have with GD. We wished to use the OCI-R obsessional thoughts subscale from the OCI-R to measure obsessional thoughts because it is a reliable tool for this [18, 58]. The full use of the OCI-R is typically used as a measure for a variety of OCD symptoms. It was therefore important that we demonstrated obsessional thoughts, and not other clinical features of OCD, are related to GD to support our decision for only using one subscale from the measure. We ran an initial hierarchical regression analysis which included all the subscales of the OCI-R as independent variables and the GIDYQ scores as the dependent variable. Please see Supplementary Material for the full results, including those for Study 2. The obsessional thoughts subscale was the only subscale to consistently predict GIDYQ scores across the samples from Study 1 and Study 2, supporting our position on focussing on obsessional thoughts and not OCD symptoms in general. The main analyses of the present study therefore only used the obsessional thoughts subscale from the OCI-R. IBM SPSS Statistics Version 29 was used for all statistical analyses.

#### Main analyses

To test the relationship obsessional thinking and autistic traits have with GD symptoms, a hierarchical regression was carried out with GIDYQ scores as the dependent variable. Model 1 included the variables age and sex assigned at birth. Model 2 included the main effect of total AQ scores. Model 3 tested the main effect of the OCI-R obsessions subscale as well as the effect all variables have when together. To test the interaction effect the total AQ scores and OCI-R obsession subscale had with the mean GIDYQ scores, a separate linear regression was then conducted. The independent variables of total AQ score and OCI-R obsessions subscales were mean-centred to reduce multicollinearity prior to the creation of the interaction terms [17, 45].

#### Outliers

Cook’s Distance indicated no participants’ data points were above 1, meaning no outliers were present in the dataset that could have affected the analysis [10].

### Results

Sex assigned at birth and age (Model 1) was a significant predictor of GIDYQ scores, *F*(2, 142) = 5.736, *p* = .004 (see Table 1). The addition of total AQ scores (Model 2) led to a statistically significant increase in ability to predict mean GIDYQ scores, *F*(1, 141) = 15.240, *p* < .001, and explained an additional 9.0% of the variance for mean GIDYQ scores. The addition of scores for the OCI-R obsessions subscale (Model 3) led to a statistically significant increase in ability to predict mean GIDYQ scores, *F*(1, 140) = 11.523, *p* < .001, and explained an additional 6.3% of the variance for mean GIDYQ scores. The full model of age, sex assigned at birth, total AQ score and scores for the OCI-R obsessions subscale to predict mean GIDYQ scores (Model 3) was statistically significant, *F*(4,140) = 10.366, *p* < .001, and explained 20.6% of the variance for mean GIDYQ scores. The interaction between total AQ scores and scores for the OCI-R obsessions subscale (see Table 2) did not have a significant effect on mean GIDYQ scores (*p* = .169).

**Table 1:** Results of the hierarchical regression with mean GIDYQ scores as the dependent variable

Predictor	*B*	*β*	*p* values	*R*^2^	Adjusted *R*^2^
Model 1				.075	.062
Age	.014	.270	**.001**		
Sex assigned at birth	− .039	− .030	.712		
Model 2				.165	.147
Age	.011	.226	**.004**		
Sex assigned at birth	− .077	− .059	.448		
AQ total score	− .020	− .305	**< .001**		
Model 3				.229	.206
Age	.007	.138	.084		
Sex assigned at birth	− .046	− .035	.639		
AQ total score	− .015	− .229	**.004**		
OCI-R obsessions subscale	− .055	− .281	**< .001**		

Bold values indicate the significant results

*AQ* Autism-Spectrum Quotient, *OCI-R* Obsessive Compulsive Inventory-Revised, *GIDYQ* Gender Identity/Dysphoria Questionnaire

**Table 2:** Results of the linear regression interaction effect with centred mean GIDYQ as the dependent variable

Predictor	*b*	*β*	*p* values	*R*^2^	Adjusted *R*^2^
Model 1				.218	.201
Total AQ score (centred)	− .017	− .250	**.002**		
OCI-R obsessions subscale (centred)	− .059	− .301	**< .001**		
Total AQ score (centred) × OCI-R obsessions subscale (centred)	− .002	− .092	.247		

Bold values indicate the significant results

### Discussion Study 1

This is the first study to show obsessional thoughts are related with traits of GD, with greater obsessional thoughts being related to greater traits of GD. The results of Study 1 also showed autistic traits are associated with traits of GD. Obsessional thoughts and autistic traits were each uniquely associated with traits of GD, as their interaction term was not significant with GIDYQ scores. Study 1 included a non-clinical sample, and so further investigation is needed with clinical samples of people diagnosed with autism, OCD and GD to provide more clinically relevant results.

## Study two

The results from Study 1 revealed that the degree of autistic traits and obsessional thoughts were each uniquely associated with greater traits of GD in a non-clinical adult sample. However, it is of interest to test if the same relationships are evident in clinical samples including autistic and OCD samples. Therefore, the main aim of Study 2 was to test these relationships in clinical samples, which included a sample of people diagnosed with either autism or OCD, and control sample with no diagnoses. Participants diagnosed with GD were also recruited to compare GD scores to clinically relevant levels. The GD scores for the groups were compared to each other. Following the results of Study 1, it was hypothesised that autistic traits and obsessional thoughts would each be uniquely associated with traits of GD in the clinical sample in general. We also hypothesised that traits of GD would be highest in the GD group, followed by the autism sample with the next highest GD scores, the OCD group having the next highest scores, and the control group having the lowest scores.

### Methods

#### Participants

A total of 347 participants were recruited for the study (mean age = 27.41, SD = 10.07; sex assigned at birth = 100 male, 247 female). Participants were eligible for the study if they either self-reported having a diagnosis of autism, OCD, or GD, or they self-reported having no psychiatric diagnoses. The final sample^3^ included 226 participants across four different groups for analyses (mean age = 28.22, SD = 11.01; sex at birth = 79 male, 147 female).

The autism group consisted of 65 participants (see Table 3). Participants were recruited using the research participant database for the Centre for Applied Autism Research at the University of X, social media (e.g., Reddit, Instagram, Twitter), and various autism-specific charities and support groups (e.g., Autistica). All participants eligible for the autism group self-reported having a formal diagnosis of autism from a qualified professional according to international criteria [3]. The mean total AQ score for the autism group (mean AQ = 35.55, SD = 7.62) was comparable to mean AQ scores reported for clinical autism samples in previous clinical studies [6, 59]. Scores for the AQ were also compared between the four diagnostic groups to support claims of an autism diagnosis. Results from a one-way ANOVA on the mean total AQ scores revealed a significant difference between the groups, *F*(3, 220) = 44.640, *p* < .001. Please see the Supplementary Materials for all post-hoc tests when comparing measure scores between the diagnostic groups.

**Table 3:** Comparison of demographics and mean scores across the groups included in Study 2

Demographics and scores	Group			
	ASD (65)	OCD (46)	GD (49)	Controls (66)
Sex ratio (male:female)	30:35	9:37	14:35	26:40
Identify as transgender (%)	15 (23.1)	4 (8.7)	45 (91.8)	3 (4.5)
Mean age (SD)	25.89 (9.29)	27.76 (10.46)	24.02 (8.27)	33.94 (12.51)
Mean AQ score (SD)	35.55 (7.62)	26.57 (9.22)	25.98 (9.85)	18.36 (7.67)
Mean OCI-R score (SD)	24.02 (12.84)	36.83 (15.12)	24.08 (14.83)	15.98 (11.51)
Mean GIDYQ score (SD)	3.92 (.66)	4.29 (.46)	3.46 (.88)	4.44 (.38)

Lower scores on the GIDYQ reflect higher GD symptoms

*AQ* Autism-Spectrum Quotient, *OCI-R* Obsessive Compulsive Inventory-Revised, *GIDYQ* Gender Identity/Dysphoria Questionnaire

The OCD group was comprised of 46 participants. Participants were recruited from social media (e.g., Reddit, Instagram, Twitter etc.) and various OCD-specific charities and support groups (e.g., OCDUK). Participants eligible for this group self-reported having a formal diagnosis of OCD provided by a qualified professional according to international criteria [3]. In addition, they completed the OCI-R as a measure of the degree of OCD symptoms to support their self-reported claims of being diagnosed with OCD. The mean total OCI-R score for the OCD group (mean OCI-R = 36.83, SD = 15.12) exceeded the mean OCI-R scores reported for clinical OCD samples in previous clinical studies [18, 28]. Results from a one-way ANOVA revealed a significant difference in mean OCI-R scores between the diagnostic groups, *F*(3, 221) = 21.784, *p* < .001. An additional one-way ANOVA revealed a significant difference in scores for the OCI-R obsessional thoughts subscale between the diagnostic groups, *F*(3, 222) = 18.287, *p* < .001.

A total of 49 participants were recruited for the GD group. These participants were recruited using social media (e.g., Reddit, Instagram, Twitter, etc.) and various transgender-specific and GD-specific charities and support groups (e.g., Mermaids). Participants for this group self-reported having a formal diagnosis of GD by a qualified professional according to international criteria [3]. Twenty-six participants reported having completed medical procedures to transition to their desired gender. The mean GIDYQ score for the GD group (mean = 3.46, SD = .88) was higher than means reported in previously published clinical GD samples [14, 46]. However, when not including those 26 participants that had medically transitioned, the GIDYQ score for the GD group (mean = 2.79, SD = .63) did match the means reported in previously published clinical GD samples. Group differences in GIDYQ scores are reported below in the main results, demonstrating that the mean GIDYQ score for the GD group was significantly lower than all the other groups (demonstrating elevated traits of GD).

A total of 66 participants were recruited for the control group. These participants were recruited using social media (e.g., Reddit, Instagram and Twitter), word of mouth in the local community, and the Undergraduate Research Participation Scheme at the University of X. All participants for the control group self-reported having no psychiatric diagnoses.

#### Procedure and measures

The measures and procedures used in Study 2 were identical to those used in Study 1 (see Methods section of Study 1 above).

#### Outliers

Cook’s Distance values were not above 1, suggesting outliers did not influence the dataset [10].

#### Data analyses

##### Hierarchical regression and interaction effect

The design of the hierarchical regression and the interaction effect were the same as those in Study 1. A hierarchical linear regression with mean GIDYQ score as the dependent variable was run using a combined sample of all participants recruited for Study 2. Model 1 included the variables of age and sex assigned at birth. Model 2 added the main effect of total AQ scores. Model 3 added the main effect of the OCI-R obsessions subscale scores. A linear regression was then conducted to analyse the interaction effect of AQ scores with OCI-R obsession subscale scores on mean GIDYQ scores. Prior to the creation of the interaction terms in the regression models, the independent variables of total AQ score and scores for the OCI-R obsessions subscale were mean centred.

#### ANCOVA

A one-way ANOVA, with significance level of *p* < .05, was used to test the mean ages between the groups and revealed a significant difference in mean ages between the diagnostic groups, *F*(3, 222) = 10.482, *p* < .001. See the Supplementary Materials for the post-hoc test results. Chi-square analysis revealed there were also significant differences in ratios of sex assigned at birth between groups, χ^2^(3, *N* = 226) = 9.827, *p* = .020. Based on the results both age and sex assigned at birth were added as covariates in the ANCOVA analyses. We tested the homogeneity of regression slopes for the two covariates to test the relationships between mean GIDYQ scores and the two covariates are the same in each diagnostic group. The assumption of homogeneity of regression slopes was not violated for neither age (*p* = .296) nor sex assigned at birth (*p* = .186), so ANCOVA was appropriate for use [17]. While Shapiro-Wilk tests revealed the GIDYQ score data for two of the groups was not normally distributed (*p* < .05), the skewness and kurtosis values were at acceptable levels to be appropriate for ANOVA analyses [7]. Therefore, an ANCOVA analysis was run to compare mean GIDYQ scores between the four diagnostic groups (autism, OCD, GD and controls). Mean GIDYQ scores were the dependent variable and diagnostic group was the independent variable, with age and sex assigned at birth as the covariates. Bonferroni corrections was used for the post hoc test.

### Results

#### Hierarchical regression of AQ total score and OCI-R subscale

Results showed that Model 1 of sex assigned at birth and age was a significant predictor of mean GIDYQ score, *F*(2, 223) = 8.668, *p* < .001 (see Table 4). The addition of total AQ scores (Model 2) led to a statistically significant increase in ability to predict mean GIDYQ scores, *F*(1, 222) = 16.057, *p* < .001, and explained an additional 6.3% of the variance for mean GIDYQ scores. The addition of scores for the OCI-R obsessions subscale (Model 3) led to a statistically significant increase in ability to predict mean GIDYQ scores, *F*(1, 221) = 10.045, *p* = .002, and explained an additional 3.8% of the variance for mean GIDYQ scores. The full model of age, sex assigned at birth, total AQ score and scores for the OCI-R obsessions subscale to predict mean GIDYQ scores (Model 3) was statistically significant, *F*(4,221) = 11.505, *p* < .001, and explained 17.2% of the variance for mean GIDYQ scores. Age (*p* = .004), total AQ score (*p* = .002) and scores for the OCI-R obsessions subscale (*p* = .002) were significant predictors of mean GIDYQ scores. The interaction between total AQ scores and scores for the OCI-R obsessions subscale (see Table 5) did not have a significant effect on mean GIDYQ scores (*p* = .169).

**Table 4:** Results of the hierarchical regression with mean GIDYQ scores as the dependent variable

Predictor	*B*	*β*	*p* values	*R*^2^	Adjusted *R*^2^
Model 1				.072	.064
Age	.017	.269	**< .001**		
Sex assigned at birth	− .001	− .000	.996		
Model 2				.135	.123
Age	.015	.225	**< .001**		
Sex assigned at birth	− .005	− .003	.960		
AQ total score	− .017	− .254	**< .001**		
Model 3				.172	.157
Age	.012	.186	**.004**		
Sex assigned at birth	− .011	− .007	.904		
AQ total score	− .014	− .204	**.002**		
OCI-R obsessions subscale	− .036	− .206	**.002**		
Model 4					
Age	.013	.194	**.002**		
Sex assigned at birth	0.12	.008	.894		

Bold values indicate the significant results

*AQ* Autism-Spectrum Quotient, *OCI-R* Obsessive Compulsive Inventory-Revised, *GIDYQ* Gender Identity/Dysphoria Questionnaire

**Table 5:** Results of the linear regression interaction effect with mean GIDYQ as the dependent variable

Predictor	*b*	*β*	*p* values	*R*^2^	Adjusted *R*^2^
Model 1				.147	.136
Total AQ score (centred)	− .016	− .244	**< .001**		
OCI-R obsessions subscale (centred)	− .040	− .232	**< .001**		
Total AQ score (centred) × OCI-R obsessions subscale (centred)	− .001	− .087	.169		

Bold values indicate the significant results

#### ANCOVA comparing mean GIDYQ scores between diagnostic groups

**Fig. 1:**
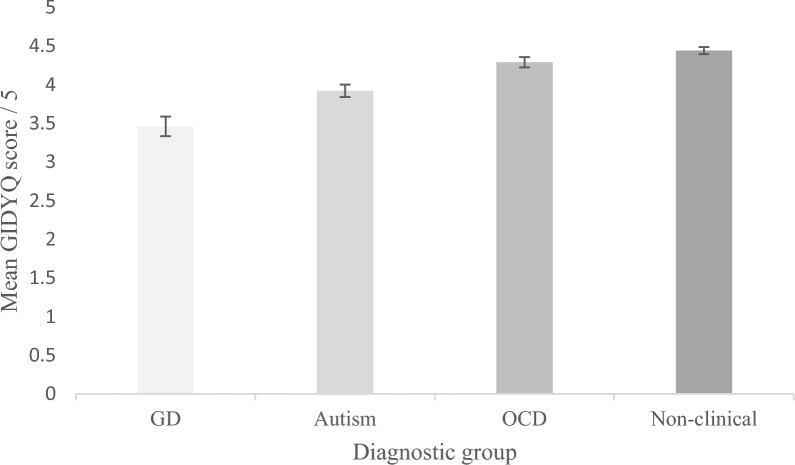
Bar graph of mean GIDYQ scores for the four groups, with lower scores indicating greater traits of GD. *Note*: *GD* gender dysphoria, *OCD* obsessive and compulsive disorder

Results showed that sex assigned at birth was not significantly related to mean GIDYQ scores, *F*(1,226) = .024, *p* = .877, partial *η*^2^ = .000. The covariate age was significantly related to mean GIDYQ score, *F*(1,226) = 4.032, *p* = .046, *η*^2^ = .018. Diagnostic group was shown to have a significant effect on mean GIDYQ score after controlling for the effect of sex assigned at birth and age, *F*(3,226) = 21.552, *p* < .001, *η*^2^ = .227. Bonferroni-corrected post-hoc tests revealed mean GIDYQ score for the GD group (*M* = 3.46, SD = .09) was significantly lower than mean GIDYQ score for the autistic group (*M* = 3.94, SD = .08, *p* < .001), the OCD group (*M* = 4.29, SD = .09, *p* < .001) and the control group (*M* = 4.40, SD = .08, *p* < .001, Fig. 1). The autistic group also had significantly lower mean GIDYQ scores when compared to the OCD group (*p* = .023) and the non-clinical group (*p* < .001). No other significant differences were reported between the diagnostic groups.

### Study 2 discussion

This is the first study to show that both obsessional thoughts and total autistic traits are uniquely associated with traits of GD in a clinical sample. Further group comparison results showed that the autistic group reported significantly greater traits of GD compared to both the OCD and control groups, but fewer traits of GD compared to the GD group who reported the highest level of GD traits. The OCD group reported greater traits of GD compared to the control group, although this difference was not statistically significant.

### General discussion

This is the first study to report a significant relationship between obsessional thoughts and greater traits of GD, which was robust as it was found across both clinical and non-clinical adult samples. In addition, the degree of autistic traits was also related to traits of GD, with greater autistic traits related to greater traits of GD across both clinical and non-clinical samples. Each factor was shown to be uniquely associated with traits of GD as no interaction effect between them was evident. Traits of GD were significantly higher in the autistic group when compared to the control group but were less than those in the clinical GD sample. Although obsessional thoughts were associated with greater GD traits across the whole sample, the OCD group did not report significantly more GD traits than the control group. Together, these findings show the degree of autistic traits and obsessional thoughts are each associated with traits of GD, which is evident in a sample from the general population as well as a clinical sample including those diagnosed with autism, OCD and GD.

While previous research has shown evidence for greater obsessional thoughts in children with GD, it has been raised that the measure of obsessions utilised in the research were reflective of more general measures of autistic traits [47], making it unclear if the relationships with obsessions simply represented more general associations between autistic traits and GD. Additional research has found similar findings [53, 61], but only a single item was used to measure obsessional thinking. Although the results were interpreted to reflect the obsessional thoughts characteristic of autism, the results did not actually provide much information about the nature of the obsessional thinking. The present study utilised a multi-item measure of obsessions as part of the full OCI-R which was developed and validated for measuring OCD symptoms in diverse clinical [18, 20] and non-clinical samples [5, 48, 60], rather than autistic traits, to test if the negative feelings associated with obsessional thoughts were associated with traits of GD in adults. The results here once again showed a relationship between obsessional thoughts and GD traits. The finding that elevated obsessional thoughts were related to greater traits of GD was robust as it was evident across separate clinical and non-clinical samples. The present results provide evidence for the association between obsessional thinking and GD that is differentiable from autistic trait measures.

The present results also provide further understanding about the characteristics of the obsessional thinking related to GD, with the OCI-R obsessions subscale including items measuring negative feelings about unpleasant thoughts and feeling unable to control them or stopping them from coming to mind. The content of the negative obsessional thoughts remains unknown as this was not measured via the items of the OCI-R obsessional thoughts subscale, as qualitative research (e.g. open-ended responses by participants) were not included in the present study. It has been proposed that negative obsessional thoughts which are focused on gender-related themes may lead to traits of GD in autistic people [53, 61]. Thoughts related to GD may then be maintained over time because of a person’s obsessional thinking style. Conversely, the relationship between obsessional thoughts and traits of GD may be due to gender related obsessional thoughts being a consequence of traits of GD. It is unclear from the results of the analysis reported here whether obsessional thoughts precipitated traits of GD, or if traits of GD gave rise to obsessional thoughts. Future longitudinal research about obsessional thoughts and traits of GD should be explored in further research to test potential causal mechanisms and about the direction of the relationship.

We also found that autistic traits were positively associated with traits of GD across clinical and non-clinical adult samples. This expands on the findings of previous research [22, 30] which had previously shown that autistic traits were correlated with traits of GD within samples of combined autistic and non-autistic adult participants. The degree of autistic traits has previously been shown to be higher in people diagnosed with autism than other clinical conditions [16, 39]. This may explain why rates of autism diagnoses are notably higher in gender clinics than those in the general population. Although the degree of autistic traits varies across people on the spectrum [25], from these results we could hypothesise that autistic people who exhibit greater degrees of autistic traits may experience elevated traits of GD compared to autistic people with lesser degrees of autistic traits.

There is debate about the extent autism and autistic traits are related with GD and transgender identities [19, 51, 52]. It has been suggested that the stigma, discrimination, and rejection experienced by transgender people will create “false” autistic traits in this population, such as social and communication difficulties [19, 51, 52]. Yet, the autistic measure used in the present study (the AQ) covers several core domains of autism, such as rigid thinking and reduced imagination as well as communication difficulties. In fact, all subscales of the AQ have been shown to be significantly correlated with traits of GD [22], demonstrating the utility of the AQ as a robust tool for measuring a range of core autistic traits and their relationship with GD. In addition, unlike previous research [47, 56], the present study was focussed on measuring the relationship between autistic traits and traits of GD across clinical and non-clinical samples, and not concerned about measuring the degree of autistic traits in trans and gender dysphoric people. Taking this into consideration, the results here contribute to the ongoing debate and show autistic traits are indeed associated with traits of GD. Further qualitative research could help to investigate about the potential factors underlying the relationship between autistic traits as measured by the AQ and GD.

We also consistently found no interaction effect between obsessional thoughts and autistic traits in their relationship with traits of GD, across clinical and non-clinical groups. This means that the two variables are independently associated with traits of GD and that their relationships with GD do not change dependent on the degrees of the other variable. A person with high obsessional thoughts may experience elevated traits of GD regardless of whether they also experience low or high degrees of autistic traits, and a person with high autistic traits may experience elevated traits of GD regardless of whether they also experience low or high degrees of obsessional thoughts. This could suggest that the two provide separate pathways to the experience of GD, but the mechanisms of these pathways remain unclear, especially when case studies have documented individuals being diagnosed with autism, OCD and gender dysphoria [24]. There are some theories on why autistic traits may lead to traits of GD, such as communication difficulties [11] and reduced Theory of Mind [30], and these may offer an explanation for why autistic people reported greater features of GD compared to OCD and control groups but were not explored in the present study. Yet, the concept and findings of obsessional thoughts being associated with traits of GD is novel to the literature. Future research should aim to uncover the content of these obsessional thoughts in relation to GD to better our current understanding of this phenomena.

To the best of the authors’ knowledge this is the first study to compare GD scores between gender dysphoric samples and other clinical conditions, with the results revealing that the GD group reported significantly more traits of GD than all the other groups as expected. Further results showed that the autism group reported elevated traits of GD compared to both the OCD and the control groups, but fewer GD traits than the GD diagnosed group. This finding is consistent with research showing that GD traits are elevated in autistic samples compared to non-autistic control groups [22, 30, 32], but they did not show the same degree of GD traits as people diagnosed with GD. The current study also further demonstrated that the autistic group showed greater GD traits than another clinical group, in this case a group with diagnosed OCD. However, since higher obsessional thoughts were associated with greater traits of GD and the OCD group had higher scores for obsessional thoughts than the other diagnostic groups, we would have expected the scores for traits of GD in the OCD group to be significantly higher compared to controls. While the OCD group had greater traits of GD compared to the controls, this difference was not statistically significant demonstrating comparable levels of GD traits between them, indicating that the presence of obsessional thoughts alone does not necessarily lead to elevated traits of GD and that the relationship between obsessional thoughts and traits of GD might be moderated by other factors present in the autistic and gender dysphoric groups.

### Limitations

There are some limitations of the study that should be noted. Although participants for the diagnostic groups in study two self-reported a diagnosis, we did not carry out diagnostic interviews to verify their diagnosis. However, the participants completed screening measures related to their diagnoses and the mean group scores on the screening measures were consistent or higher than scores reported for clinical samples in previous studies using each respective measure. Group comparisons also showed that the scores for each group on their respective screening measures were significantly higher compared to all the other groups (see Participants section). However, it is worth noting that measures of mental health symptoms, such as anxiety or depression, were not completed by participants. This may have influenced the pattern of results observed because anxiety and depression are highly prevalent in autistic people [35]. An additional limitation to the study is that self-reported GD symptoms were measured at just one point in time. GD is reported to be a fluctuating and continuous construct [12, 44], meaning the degree of GD symptoms reported by participants may have changed over time. Finally, while we draw conclusions that the obsessional thoughts which are associated with traits of GD involve unpleasant thoughts and feeling unable to control them or stopping them from coming to mind, the true nature of the content of these thoughts cannot be measured by the measures administered here. This information can only be obtained through the use of measures which are more specific in their questioning, or through cognitive interviewing. Future research is needed to provide a more definitive insight into the nature of the obsessional thoughts in relation to traits of GD.

## Conclusion

The findings here are the first to report that obsessional thoughts and total autistic traits are each uniquely associated with GD traits in both clinical and non-clinical adult samples. These findings are consistent with a growing body of research reporting a strong relationship between autism and GD and extend the results to show that obsessional thoughts characteristic of OCD also positively relate to GD traits in adults. The results from Study 1 demonstrated these effects in a non-clinical sample, showing the relationships exist with subclinical levels of these traits. Study 2 showed the effects in a clinical sample including people diagnosed with autism, OCD and GD, demonstrating once again that obsessions involving negative feelings and being unable to control them were related to greater GD traits. However, the present research did not reveal about the contents of the obsessional thoughts associated with GD traits, so future research should explore more about these topics.

## Supplementary Information

Additional file 1.

## Data Availability

The data for this paper is secured by the University of Bath and is available upon request to the corresponding author.

## Abbreviations

AQ: Autism Quotient
GD: Gender dysphoria
GIDYQ: Gender Identity/Dysphoria Questionnaire
OCD: Obsessive compulsive disorder

## Footnotes

1 A total of 42 participants did not complete above 50% of the survey and were excluded from the study.

2 Results of the analysis did not change substantially when transgender participants were removed, so the 14 transgender participants were included in the main analyses.

3 A total of 17 participants were excluded for having comorbid diagnoses with combinations of autism, OCD or GD, and 104 participants were excluded for reporting having different clinical diagnosis to autism, OCD or GD (e.g., schizophrenia or PTSD).
