# Supplementary material for: Obsessional thinking and autistic traits are each uniquely associated with greater traits of gender dysphoria in clinical and nonclinical adult samples
Source: Mol Autism. 2025 Mar 17;16:20. doi: 10.1186/s13229-025-00649-1 (PMC11916952; doi:10.1186/s13229-025-00649-1)
Supplement: Supplementary file 1 — Additional file 1. [file 13229_2025_649_MOESM1_ESM.pdf]

Supplementary material

One of the primary aims for the present study was to look into the relationship non-autistic obsessional thoughts have with feelings of gender dysphoria (GD). Research has shown obsessional thoughts to be related to feelings of GD (e.g., Zucker et al., 2017), and there is currently no research to support the idea that OCD symptoms in general are related to feelings of GD. The OCI-R is a validated measure for OCD symptoms in general, and its subscale 'obsessional thinking' has also been shown to have good internal consistency, ranging from .77 - .84 (Hajcak et al., 2004; Williams et al., 2013). Initial hierarchical regression analyses were run to demonstrate that non-autistic obsessional thoughts, and not other clinical features of OCD, are related to GD to support our decision for only using one subscale from the OCI-R measure in our main analyses. Separate regressions were conducted for the samples of Study 1 and Study 2. For each regression, mean GIDYQ scores were used as the dependent variable. Model 1 included participants' age and sex assigned at birth and Model 2 included the six subscales of the OCI-R.

Study 1

Model 1 showed age and sex assigned at birth significantly predicted mean GIDYQ scores, $F(2, 142) = 5.736$, $p = .004$, and explained 7.5% of the variance. The addition of the OCI-R subscales (Model 2) led to a statistically significant increase in ability to predict mean GIDYQ scores, $F(6, 136) = 4.013$, $p = .001$, and explained 13.9% of the variance but only the obsessions subscale significantly predicted mean GIDYQ scores ($p = .008$). **See Table 1.**

Table 1. Results of the hierarchical regression for OCI-R subscales predicting mean GIDYQ scores for samples from Study 1.

Predictor	*b*	*β*	*p values*	R^2	*Adjusted* R^2
Model 1				.075	.062
Age	.014	.270	**.001**		
Sex assigned at birth	-.039	-.030	.712		
Model 2				.214	.168
Age	.008	.165	.053		
Sex assigned at birth	-.030	-.023	.772		
OCI-R obsessions subscale	-.049	-.251	**.008**		
OCI-R washing subscale	-.001	-.002	.983		
OCI-R hoarding subscale	-.043	-.200	.053		
OCI-R ordering subscale	.002	.008	.934		
OCI-R checking subscale	-.012	-.047	.628		
OCI-R neutralising subscale	-.022	.097	.293		

Study 2

Model 1 showed age and sex assigned at birth significantly predicted mean GIDYQ scores, $F(2, 223) = 8.668$, $p < .001$, and explained 7.2% of the variance. The addition of the OCI-R subscales (Model 2) led to a statistically significant increase in ability to predict mean GIDYQ scores, $F(6, 217) = 5.079$, $p < .001$, and explained 11.4% of the variance. Only the obsessions subscale ($p < .001$) and the OCI checking subscale significantly predicted mean GIDYQ scores ($p = .004$). **See Table 2.**

Table 2. Results of the hierarchical regression for OCI-R subscales predicting mean GIDYQ scores for samples from Study 2.

Predictor	*b*	*β*	*p values*	R^2	*Adjusted* R^2
Model 1				.072	.064
Age	.017	.269	**< .001**		
Sex assigned at birth	-.001	-.000	.996		
Model 2				.186	.156
Age	.013	.206	.002		
Sex assigned at birth	.042	.028	.652		
OCI-R obsessions subscale	-.053	-.306	**< .001**		
OCI-R washing subscale	.020	.089	.238		
OCI-R hoarding subscale	-.025	-.122	.091		
OCI-R ordering subscale	-.012	-.064	.400		
OCI-R checking subscale	.059	.262	.004		
OCI-R neutralising subscale	-.027	-.127	.139		

To validate self-reported claims of GD, OCD and autism diagnoses, participants were asked to complete the AQ (Baron-Cohen et al., 2001), the OCI-R (Foa et al., 2002), and the GIDYQ (Deogracias et al. 2007). Scores for these measures were then compared between the four diagnostic groups (GD, autism, OCD, and controls).

Bivariate correlations between the AQ total score, the OCI-R obsessional thoughts subscale, and GIDYQ scores.

The results from Study 1 found AQ total scores to be significantly correlated with the OCI-R obsessional thoughts subscale (r = .304, p < .001), and mean GIDYQ score (r = -.331, p < .001). The OCI-R obsessional thoughts subscale was also significantly correlated with mean GIDYQ score (r = -.403, p < .001).

Study 2 post-hoc tests when comparing scores on the AQ and OCI-R between diagnostic groups.

AQ scores

Tukey post-hoc tests showed that the mean total AQ score for the autism group (M = 35.55, SD = 7.66) was significantly higher compared to the GD group (M = 25.98, SD = 9.85, p < .001), the OCD group (M = 26.57, SD = 9.22, p < .001) and the control group (M = 18.36, SD = 7.67, p < .001).

OCI-R mean score

Tukey post-hoc tests revealed the mean OCI-R score for the OCD group (M = 36.83, SD = 15.12) was significantly higher compared to the autism group (M = 24.02, SD = 12.84, p < .001), the control group (M = 15.98, SD = 11.51, p < .001), and the GD group (M = 24.08, SD = 14.84, p <.001).

OCI-R obsessional thoughts subscale

Tukey post-hoc tests revealed scores for the OCI-R obsessional thoughts subscale for the OCD group (M = 8.50, SD = 3.73) was significantly higher compared to the autism group (M = 5.37, SD = 3.87, p < .001), the control group (M = 3.39, SD = 3.04, p < .001), and the GD group (M = 6.45, SD = 4.17, p = .036).

Post hoc test results when comparing mean ages between the diagnostic groups

Tukey post-hoc tests revealed the mean age for the control group (mean = 33.94, SD = 12.51) was significantly higher than the GD group (mean = 24.02, SD = 8.27, p < .001), the autistic group (mean = 25.89, SD = 9.29, p < .001) and the OCD group (mean = 27.76, SD = 10.46, p = .012), but there were no other significant differences between the groups.

References:

Baron-Cohen, S., Wheelwright, S., Skinner, R., Martin, J., & Clubley, E. (2001). The autism-spectrum quotient (AQ): evidence from Asperger syndrome/high-functioning autism, males and females, scientists and mathematicians. *Journal of Autism and Developmental Disorders, 31*(1), 5–17. https://doi.org/10.1023/a:1005653411471

Deogracias, J. J., Johnson, L. L., Meyer-Bahlburg, H. F., Kessler, S. J., Schober, J. M., & Zucker, K. J. (2007). The gender identity/gender dysphoria questionnaire for adolescents and adults. *Journal of Sex Research, 44,* 370 – 379. http://dx.doi.org/10.1080/00224490701586730

Foa, E. B., Huppert, J. D., Leiberg, S., Langner, R., Kichic, R., Hajcak, G., & Salkovskis, P. M. (2002). The obsessive-compulsive inventory: Development and validation of a short version. *Psychological assessment, 14*(4), 485–496. https://pubmed.ncbi.nlm.nih.gov/12501574/

Hajcak, G., Huppert, J. D., Simons, R. F., & Foa, E. B. (2004). Psychometric properties of the OCI-R in a college sample. *Behavior Research and Therapy, 42*(1), 115–123. https://doi.org/10.1016/j.brat.2003.08.002

Williams, M., Davis, D.M., Thibodeau, M.A., & Bach, N. (2013). Psychometric properties of the obsessive-compulsive inventory revised in African Americans with and without obsessive-compulsive disorder. *Journal of Obsessive-Compulsive and Related Disorders, 2*(4), 399-405. https://doi.org/10.1016/j.jocrd.2013.07.003

Zucker, J. K.., Nabbijohn, A. N., Santarossa, A., Wood, H., Bradley, S. J., Matthews, J., & VanderLaan, D. P. (2017). Intense/obsessional interests in children with gender dysphoria: a cross-validation study using the Teacher's Report Form. *Child and Adolescent Psychiatry and Mental Health, 11*:51. doi: 10.1186/s13034-017-0189-9
